# Unexpected Positive Cultures After Failed Proximal Humerus Osteosynthesis: Why a Two-Stage Procedure Could Be Safer

**DOI:** 10.3390/jcm15083162

**Published:** 2026-04-21

**Authors:** Raffaele Garofalo, Nunzio Lassandro, Angelo De Crescenzo, Riccardo Ranieri, Angelo Del Buono, Alberto Fontanarosa

**Affiliations:** 1 IRCCS Humanitas Reserch Hospital, Humanitas University, 20089 Milan, Italy; raffaele.garofalo@hunimed.eu (R.G.); r.ranieri@castmed.it (R.R.); 2 Ecclesiastical Entity Regional General Hospital “F. Miulli”, 70021 Acquaviva delle Fonti, BA, Italy; nunzio.lassandro@miulli.it (N.L.); a.decrescnzo@miulli.it (A.D.C.); 3 Villa Lucia Hospital, 70014 Conversano, BA, Italy; angelo_delbuono@libero.it

**Keywords:** unexpected positive culture, humerus fractures, osteosynthesis, infection

## Abstract

**Background**: Treatment of failed osteosynthesis of fractures of the proximal humerus with one-stage or two-stage surgery is difficult and clinical results are poor. The aim of this work is to evaluate the microbiological positivity of devices removed due to osteosynthesis failure. Furthermore, the clinical outcomes of these patients were evaluated at a follow-up of minimum 6 months, to assess the recovery of range of motion and the reduction in pain. **Methods**: A retrospective analysis was performed on 15 patients treated from September 2021 to September 2023 for failure of previous proximal humerus synthesis. These treatments included implant removal and arthrolysis. None of these patients showed signs of infection. Demographic data, VAS, ASES, Constant score, and range of motion (ROM) were assessed before surgery and at least 6 months of follow-up. Removed devices were processed in MicroDTTect^®^ system, to increase the sensitivity of microbiological cultures. The cultural and clinical results of device removal surgery were analyzed. **Results**: Culture results were positive in eight out of 15 patients. Slow-growing anaerobic bacteria were the most isolated microorganisms, particularly *C. acnes* (62.5%). Improvement in patients’ passive ROM was observed. The patients went from a preoperative VAS of 8.4 (±1.1) to a VAS of 2 (±1.1) at follow-up. Similarly, we observed an increase in ASES from 9 ± 6 to 50.2 ± 2.3 and Constant score from 17 (15–18) to 40.7 ± 3.3 at a follow-up of at least 6 months. **Conclusions**: Two-stage procedure should always be considered in the context of proximal humerus synthesis failure. Arthrolysis with postoperative physiotherapy prepares the shoulder for definitive prosthesis implantation.

## 1. Introduction

Proximal humerus fractures are among the three leading causes of fractures in adults, and with the increase in the average age of the global population, a progressive increase in these fractures has been observed [1].

The treatment is controversial, debated, and not well defined yet [2,3]. Reduction and osteosynthesis of proximal humeral epiphysis leads to up to 48% of complication rates [4,5], and revision surgery is needed in almost 15% of patients, mostly those older than 60 years [6,7].

In revision surgery, removal of the means of synthesis, with or without arthrolysis, and reverse shoulder arthroplasty are usually advocated. In this case, reverse shoulder arthroplasty is associated with an increased infection rate, estimated at around 11–12% [8,9]. Therefore, in elderly patients with 3–4 fragment fractures, the main trend is to perform reverse shoulder arthroplasty as a first-line treatment [10].

On biological samples, bacterial growths may be indexed for unexpected positive cultures (UPCs), a condition in which the bacterium is present without any clinical infection evidence. UPCs are very frequent, both in primary and secondary shoulder surgery [11,12,13,14].

Nevertheless, after removal of fixation means and one-stage shoulder arthroplasty, even if the bacterium is isolated intraoperatively, patients may not develop an infection over time [11].

There is evidence that, when removing plating from the clavicle [12] or humerus [13], unexpected positive cultures (UPCs) may be detected in most patients, 82% and 50%, respectively, usually caused by the *C. acnes*. Interestingly, in surgical samples, UPCs are more likely associated with shoulder stiffness [13].

*C. acnes* is a slow-growing Gram-positive anaerobic bacillus which is frequently isolated from specimens taken during primary shoulder surgery, both open and arthroscopic [14,15,16,17].

*C. acnes*, isolated both in tissues and intracellular structures, is associated with chronic inflammation, with increased levels of IFN-1 produced by macrophages [18], and in revision arthroscopy, with shoulder stiffness and pain [19]. Likewise, in revision arthroplasty, it is associated with osteolysis, glenoid wear, and membrane formation [20].

This study aims to assess the occurrence of UPCs after surgical revision, secondary-to-previous osteosynthesis, the influence of UPCs on preoperative clinical status, and whether arthrolysis may improve postoperative ROM.

## 2. Materials and Methods

### 2.1. Patient Selection

This is a retrospective study based on prospectively collected data, conducted on 15 patients (9 men and 6 women), who underwent removal of plating and nailing after fixation of proximal humeral fractures, between September 2021 and September 2023. All subjects were informed about the procedure, purpose of the study, and any known risks, and all provided written informed consent. The ethics committee of the Miulli Hospital approved all procedures performed (n. 6745). The main inclusion criteria were persistent shoulder pain and stiffness after plating or nailing for proximal humeral fractures. Shoulder stiffness was defined in patients with abduction reduction <90° and rotation reduction <60° after at least 6 months of postoperative physical therapy [21].

Obvious signs of acute or chronic infection were exclusion criteria. In particular, a post-operative shoulder infection was ruled out if the patient showed no signs of local inflammation, the scar was intact, and there were no fistulas. Furthermore, blood tests revealed no increase in white blood cell count, whilst the ESR and CRP were within normal limits.

### 2.2. Assessment

Two authors, both with at least 10 years of experience in shoulder surgery (AF, ADC), examined all patients preoperatively, taking account of clinical examination and radiographic findings. Patient-specific information such as age and gender at the time of surgery, clinical manifestation, preoperative range of motion, Visual Analog Scale (VAS) score, American Shoulder and Elbow score (ASES), and Constant-Murley score (CMS) were recorded for all patients [22,23,24].

Grashey, Neer, and Bernageau shoulder X-ray views and CT scanning were performed in all patients to assess avascular necrosis, nonunion, joint screw prominence, and chondral damage.

Pre-operatively, data on serum C-reactive protein (CRP), erythrocyte sedimentation rate (ESR), and white blood cell (WBC) count were also recorded.

### 2.3. Surgery

A daily application of benzoyl peroxide was used 3 days before surgery [25]. Before surgery, the skin was cleaned twice: during patient positioning, by using 3% hydrogen peroxide above the skin [26] and, then, before applying sterile draping, by using 2% chlorhexidine digluconate and 70% isopropyl alcohol solution (Lombardia H S.r.l., Albairante, Milan, Italy).

**Figure 1:**
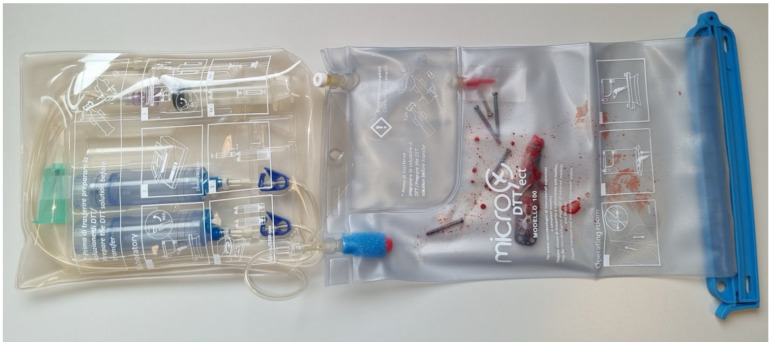
Example of implant removed and inserted in MicroDTTect bag.

All procedures were performed under regional block and general anesthesia. A single surgeon (RG) performed all surgical procedures. Patients were placed in the beach-chair position. The blade used for skin incision was replaced with an uncontaminated blade. After dissection, the plate or nail was removed with a dedicated toolset, taking care to avoid the contact between the synthetic implant and the gloves of the surgeon. The devices were aseptically removed and immediately placed into the MicroDTTect collection system (Figure 1). Antibiotic prophylaxis with 2 g of Cefazolin was administered after device removal.

Once the fixation devices had been removed, arthrolysis surgery was performed in all instances. Specifically, all scaring tissues, fibrosis, and adhesions were excised, paying attention to surrounding structures, mostly to the subacromial and subdeltoid spaces, and, anteriorly, to the pectoralis major muscle. In addition, rotator interval release and long head biceps tenotomy were also performed. In patients who underwent antibiotic spacer implantation for subsequent reverse prosthesis application, subscapularis muscle peeling was performed. Subsequent suturing of the subscapularis muscle remnant was performed using 2 PDS suture.

### 2.4. Bacteria Identification

The MicroDTTect procedure was performed according to the manufacturer’s instructions [27,28]. This is a closed sterile system which contains a specific concentration of dithiotreitol (DTT) to remove bacteria from the biofilm adhering to device surfaces. The samples were cultured for a maximum of 2 weeks.

In the event of a positive UPC result, a consultation with an infectious disease specialist was requested and, if deemed appropriate, antibiotic therapy was initiated.

### 2.5. Follow-Up

All patients were followed up with clinical assessment up to six months.

The intra-operative and post-operative data were recorded, including operation time (minutes), use of analgesics, and wound healing time (weeks). Post-operative stiffness [24] was also considered. Post-operative complications such as wound infection and neurological and vascular disorders were also recorded. Two orthopedic surgeons (AF, ADC) examined all the patients. VAS, ASES, and Constant-Murley scores were administered at the three month and at minimun 6 months follow-up.

### 2.6. Statistical Analysis

Mean, median, SD, and 95% confidence intervals were calculated. Categorical variables were described as frequency and percentage. Student’s *t*-test or non-parametric Mann–Whitney, as appropriate, were used to compare continuous variables. Comparisons within groups were performed with Student’s *t*-test for paired samples. Associations between categorical data were evaluated by Fisher’s exact test. A *p*-value < 0.05 was considered statistically significant. All analyses were conducted using STATA software, version 16 (Stata-Corp LP, College Station, TX, USA).

## 3. Results

The average age of the patients at the time of removal of the fixation devices was 70 (range from 59 to 73). The average time between primary reduction and fixation and revision surgery was 21 months (range from 10 to 29) (Table 1). All patients had sustained a fracture of the proximal humerus, which was treated with plate fixation in 11 cases and with an intramedullary nail in four cases.

Before revision surgery, CT and radiographic imaging revealed malunion in four patients, loss of reduction in three patients, osteonecrosis in four patients (with humeral head perforation in two patients), pseudoarthrosis in one patient, and a combination of causes in three patients.

In all patients prior to surgery, CRP and ESR levels were within the normal range, at less than 0.5 mg/dL and 10 mm/hour, respectively.

The MicroDTTect system (Figure 1) showed bacterial growth in eight out of 15 patients (53.3%). Specifically, as shown in Table 2, slow-growing anaerobic bacteria were isolated in six patients (75%): *Cutibacterium acnes* in five patients and *Peptoniphylus asaccharolyticus* in one. *P. asaccharolyticus* is a Gram-positive anaerobic coccus that has usually been identified as a commensal of the skin or in polymicrobial colonization of chronic wounds and ulcers [29]. Coagulase-negative staphylococci were identified in two patients (25%) (Table 2).

Patients with positive UPCs had greater preoperative stiffness, with passive forward flexion less than 40° and passive abduction less than 30° compared to patients without any evidence of UPCs (Table 3).

Preoperatively, VAS values, ASES, and Constant Murley scores were not significantly different comparing patients with and without UPCs (Table 4). On the other hand, postoperatively, a significant improvement in VAS, ASES, and Constant Murley scores was observed, with no inter-group differences (Table 4).

Of the 15 patients who underwent removal of fixation devices and arthrolysis, five patients had an antibiotic spacer implantation. Subsequently, all five patients with an antibiotic spacer underwent a second stage reverse shoulder arthroplasty (RSA).

Conversely, none of the 10 patients who underwent removal of fixation devices and arthrolysis without spacer implantation subsequently received a second stage RSA.

No patient developed signs of joint infection at a minimum follow-up of 6 months after removal of the fixation devices.

## 4. Discussion

Our case series is the first attempt to identify the presence of UPCs in patients undergoing revision surgery for failed osteosynthesis, using a specific device (MicroDTTect) containing DTT, with antibiofilm activity. In particular, the use of this device not only allows us to increase the accuracy of the cultures on the sampled material, but also reduces the risk of contamination of the samples taken.

Usually, physical and chemical sonication mechanisms [17,30] are used to recognize peri-implantation infections. In these cases, the biofilm-disintegrating activity of the device allows bacteria to be released and identified. In our series of patients, UPCs was detected in 53.3% of patients, despite efforts to reduce the risk of contamination of the samples examined [17].

In revision surgery, following failure of osteosynthesis, the identification of UPCs is relevant for determining the therapeutic strategy, with a significant impact on clinical outcomes. At times, the diagnosis of infection is clear, with certain clinical signs indicative of infection: a fistula, warm and painful joint, and laboratory values. However, in most cases, especially in late infections, the diagnosis may be uncertain and patients may present only with shoulder pain and stiffness [31]. In such cases, diagnosis is only possible when the bacterium is identified in tissue samples taken during surgery.

When osteosynthesis fails, a two-stage procedure is safer compared to a one-stage procedure. First of all, removal of the fixation device, combined with arthrolysis, can provide good functional outcomes [24]. Specifically, arthrolysis, when combined with fixation device removal and postoperative physiotherapy, significantly improves ROM and functional outcomes, even in patients undergoing RTSA as second stage.

In a study by Katthagen et al. [21], 45 patients underwent removal of fixation devices and arthroscopic arthrolysis due to failure of previous osteosynthesis. At 2 years, 37 (82%) patients were satisfied with the procedure, and only three patients (7%) had undergone secondary shoulder arthroplasty. Furthermore, the first surgery to remove synthetic media could allow the treatment of associated pathologies, such as rotator cuff tears, LHB tears, and malunion of tuberosities.

When fracture fixation fails, shoulder arthroplasty is very complex, with high complication rates, even in specialized centers [9,32].

In a study by Raiss et al. [8], 32 patients, who underwent reverse shoulder arthroplasty for nonunion of the surgical neck of the humerus, were evaluated at an average follow-up of 4 years. Although patients showed significant clinical improvement after RTSA surgery, a complication rate of 41% (13 patients) was observed. Specifically, dislocation of the prosthesis was the main complication, and revision arthroplasty was needed in 28% (nine patients) of cases.

In a similar study, Kilic et al. [32] reported an infection rate of 11% in a series of patients undergoing reverse shoulder arthroplasty secondary to primary osteosynthesis.

When comparing outcomes after primary RSTA versus secondary RTSA (salvage procedure after failure of osteosynthesis), Shannon et al. [33] observed no clinical differences in terms of ASES and ROM values, but higher complication rates after secondary RTSA (8% vs. 5%).

On this basis, we underline that, when osteosynthesis fails, the risk of infection increases significantly in one-stage shoulder arthroplasty. The increased risk of infections is linked to increased surgical times and the high rate of UPCs, which has been observed in patients whose implants have been removed and analyzed for bacteria [12,13]. Some articles show a high prevalence of periprosthetic infection in patients requiring secondary arthroplasty due to failure of fracture fixation [9,10]. Martinez et al. [9] reported, in 18 patients with proximal humeral non-unions treated with a reverse shoulder arthroplasty, an infection rate of 11%.

Raiss et al. [8], in their series of 32 patients treated for nonunion of the proximal humerus, observed the development of periprosthetic infection in four patients (12%). All these patients had a history of open reduction and internal fixation, but not an infection. Furthermore, it is not very clear whether the authors performed, pre- or intraoperatively, any cultural testing.

The diagnosis and management of periprosthetic infections are complex. The incidence after primary RTSA is significantly lower than that reported after secondary RTSA (after failure of osteosynthesis), ranging from 1 to 4% [34,35,36].

In this study, we observed a high rate of UPCs on the collected synthesis devices, even though all procedures were used to reduce bacterial contamination. We now point out that, given this relatively high risk of contamination, to best reduce the risk of periprosthetic infections, a two-stage shoulder arthroplasty should be recommended when primary fixation fails.

Furthermore, in our study, we observed increased preoperative stiffness in patients with positive UPCs in intraoperative specimens. A state of chronic inflammation induced by the tissue and intracellular presence of slow-growing bacteria may be responsible for greater preoperative stiffness in these patients [18].

On the other hand, in terms of pain, any significant difference was found comparing patients with positive and negative UPCs, probably in relation to the small sample size of the study group.

This study has several strengths. None of the patients was lost to the follow-up; a single experienced fellowship-trained trauma surgeon performed all surgical procedures; two independent investigators not involved in the index surgery examined the patients at follow-up; and patients were enrolled according to strict selection criteria.

The main limitations of the present study are its retrospective nature, the relatively small sample size, and the short follow-up.

We cannot exclude the possibility that the presence of UPCs may be due to contamination of the explanted material during surgery. Nevertheless, all procedures were used to limit contamination, including the use of benzoic acid and preoperative hydrogen peroxide. Furthermore, the use of the MicroDTTect device reduces the risk of contamination of the explanted material during its manipulation.

We are aware that this a preliminary study on patients candidate to reverse shoulder arthroplasty, but all these patients are objects of ongoing, longer follow-up studies.

## 5. Conclusions

Two-stage revision surgery after failed osteosynthesis allows us to identify UPCs, in which slow-growing bacteria are involved. Compared to one-stage shoulder arthroplasty, the primary removal of fixation devices and arthrolysis may improve range of motion and clinical and functional outcomes, whilst reducing the risk of infection.

## Figures and Tables

**Table 1:** Demographic data.

		All Patients	Non-UPCs	UPCs	*p*-Value
		n = 15			
**GENDER**	**FEMALES**	6 (40%)	4 (57%)	2 (25%)	0.592
	**MALES**	9 (60%)	3 (43%)	6 (75%)	
**AGE**		70 (59–73)	68 (56–73)	71 (59–77)	0.442
**Time between synthesis and revision surgery (months)**		21 ± 21	17 ± 21	24 ± 23	0.568

**Table 2:** Culture results.

Bacteria	%
**Negative cultures**	7 (46.6%)
**Positive cultures**	8 (53.3%)
***C. acnes***	5 (62.5%)
***P. asaccharolyticus***	1 (12.5%)
**Coagulase-negative staphilococci (*S. warneri*)**	2 (25%)

**Table 3:** Pre-operative passive range of motion. (° = degree; L5 = fifth lumbar vertebra; L3 = third lumbar vertebra).

Pre-Op Passive Range of Motion (°)		All Patients	Non-UPC	UPC	*p*-Value
**Anterior elevation**		93 (85–130)	130 (100–150)	90 (85–90)	0.020
**Lateral abduction**		85 (80–110)	110 (80–120)	80 (80–90)	0.077
**External rotation**		10 (0–20)	10 (10–20)	10 (0–20)	0.511
**Internal rotation**	Trochanter	6 (43%)	2 (29%)	4 (57%)	0.592
	Sacrum	3 (21%)	0 (0%)	3 (43%)	0.192
	L5	3 (21%)	3 (43%)	0 (0%)	0.192
	L3	2 (14%)	2 (29%)	0 (0%)	0.462

**Table 4:** VAS values, American Shoulder and Elbow score (ASES) values, and Constant-Murley values.

	All Patients	Non-UPC	UPC	*p*-Value
**VAS PRE-OP**	8.4 ± 1.1	8.1 ± 1.1	8.7 ± 1.1	0.347
**VAS POST-OP**	2.0 ± 1.1	2.0 ± 1.3	2.0 ± 1.0	1.000
**VAS POST-PRE**	−6.4 ± 1.5	−6.1 ± 1.5	−6.7 ± 1.6	0.499
**ASES PRE-OP**	9 ± 6	11 ± 6	7 ± 7	0.339
**ASES POST-OP**	50.2 ± 2.3	49.7 ± 1.8	50.7 ± 2.9	0.474
**ASES POST-PRE**	41 ± 6	39 ± 5	43 ± 8	0.228
**Constant PRE-OP**	17 (15–18)	17 (12–18)	17 (15–20)	0.605
**Constant POST-OP**	40.7 ± 3.3	40.6 ± 3.6	40.9 ± 3.4	0.880
**Constant POST-PRE**	25 ± 6	26 ± 8	24 ± 5	0.531

## Data Availability

The original contributions presented in this study are included in the article. Further inquiries can be directed to the corresponding author.
